# One Ferroptosis-Related Gene-Pair Signature Serves as an Original Prognostic Biomarker in Lung Adenocarcinoma

**DOI:** 10.3389/fgene.2022.841712

**Published:** 2022-03-16

**Authors:** Lei Li, Buhai Wang

**Affiliations:** ^1^ Department of Oncology, Northern Jiangsu People’s Hospital, Yangzhou, China; ^2^ Graduate School, Dalian Medical University, Dalian, China

**Keywords:** ferroptosis, lung adenocarcinoma, gene pair, prognostic marker, Cox model

## Abstract

Lung adenocarcinoma is the most common histological subtype of lung cancer which causes the largest number of deaths worldwide. Exploring reliable prognostic biomarkers based on biological behaviors and molecular mechanisms is essential for predicting prognosis and individualized treatment strategies. Ferroptosis is a recently discovered type of regulated cell death. We downloaded ferroptosis-related genes from the literature and collected transcriptome profiles of lung adenocarcinoma from The Cancer Genome Atlas (TCGA) and Gene Expression Omnibus (GEO) to construct ferroptosis-related gene-pair matrixes. Then, we performed the least absolute shrinkage and selection operator regression to build our prognostic ferroptosis-related gene-pair index (FRGPI) in TCGA training matrix. Our study validated FRGPI through ROC curves, Kaplan–Meier methods, and Cox hazard analyses in TCGA and GEO cohorts. The optimal cut-off 0.081 stratified patients into low- and high-FRGPI groups. Also, the low-FRGPI group had a significantly better prognosis than the high-FRGPI group. For further study, we analyzed differentially expressed ferroptosis-related genes between high- and low-FRGPI groups. Gene set enrichment analysis (GSEA) enrichment maps indicated that “cell cycle,” “DNA replication,” “proteasome,” and “the p53 signaling pathway” were significantly enriched in the high-FRGPI group. The high-FRGPI group also presented higher infiltration of M1 macrophages. Meanwhile, there were few differences in adaptive immune responses between high- and low-FRGPI groups. In conclusion, FRGPI was an independent prognostic biomarker which might be beneficial for guiding individualized tumor therapy.

## Introduction

Lung cancer is the second most frequent cancer and causes the largest number of deaths worldwide, acccounting to 11.4 percent of new cases and 18 percent of cancer-related deaths in 2020 (Ferlay et al., 2020). Among the subtypes of lung cancer, non-small-cell lung cancer (NSCLC) accounts for the largest part and occupies 85 percent of lung cancer cases. Specifically, lung adenocarcinoma of NSCLC, representing 60 percent, is the most common histological subtype (Arbour and Riely, 2019). Smoking is the acknowledged main risk factor for lung cancer, nevertheless, more possibly leading to squamous carcinoma than adenocarcinoma.

On the basis of histological types, clinical stages, and genetic alterations, integrative treatments are essential for NSCLC. Surgery is the first choice for localized stage I/II/IIIA/IIIB (T_3_N_2_M_0_) NSCLC. Radiotherapy achieves curative intents for people who are not eligible for surgery and is helpful for symptomatic relief in smaller doses. Platinum-based chemotherapy, a traditional non-surgical treatment, is still a choice of first line in advanced NSCLC. Due to increasing molecular targets investigated, targeted therapy is a preferred treatment for stage IV NSCLC, especially adenocarcinoma. Meanwhile, immune checkpoint inhibitors are newly regarded as second-line therapy in advanced NSCLC (Zhai et al., 2020).

Localized, regional, and metastatic NSCLC, respectively, represent 63%, 35%, and 7% 5-year survival rate (Howlader et al., 2020, based on November 2019 SEER data submission, posted on the SEER website, April 2020). More accurate and noninvasive prognostic biomarkers are needed. Exploring prognostic biomarkers based on biological and molecular mechanisms is essential for individualized treatment strategies.

Ferroptosis is a newly found type of regulated cell death (RCD), distinct from apoptosis, necroptosis, and pyroptosis (Dixon et al., 2012). Ferroptosis is a reactive oxygen species (ROS)-inducing cell death form and exhibits two main biochemical processes, ferrous iron accumulation and lipid peroxidation (Homma et al., 2019). Excessive ferrous iron (Fe^2+^), with hydrogen peroxide, generates hydroxyl radicals through the Fenton reaction and then reacts with polyunsaturated fatty acids (PUFAs) to induce lipid peroxidation. Lipid peroxidation ultimately causes membrane oxidative damage to accomplish ferroptosis.

Ferroptosis occurs through two typical pathways, the transporter-dependent pathway and the enzyme-regulated pathway (Tang et al., 2021). System X_c_^-^, composed of solute carrier family 7 member 11 (SLC7A11) and solute carrier family 3 member 2 (SLC3A2), uptakes cystine to sustain glutathione (GSH) production. Glutathione peroxidase 4 (GPX4) acts as a ferroptosis repressor and reduces lipid peroxidation while converting GSH to oxidized glutathione (GSSG).

Recently, there is growing evidence that oncology patients benefit from triggering ferroptosis of cancer cells during traditional treatments (Dixon et al., 2012; Wu et al., 2020). Classic ferroptosis inducers (FINs), such as erastin, sorafenib, cisplatin, RSL3, and FIN56, inhibit SLC7A11 activity, deplete GSH, or inhibit GPX4 activity to promote ferroptosis. Triggering ferroptosis in cancer shows the drug-resistance reversal effect and the synergistic sensitization effect with chemotherapy, target therapy, radiotherapy, and immunotherapy (Wu et al., 2020).

Several ferroptosis-related gene prognostic models were detected in multiple types of cancer. In our research, based on patients with lung adenocarcinoma, we focused on molecular mechanisms and signaling pathways of ferroptosis. Utilizing ferroptosis-related genes to build ferroptosis-related gene pairs (FRGPs) instead of single genes, we finally constructed an individualized prognostic signature biomarker of lung adenocarcinoma. On this basis, we stratified the risk of lung adenocarcinoma patients to predict prognoses and explore therapies.

## Materials and Methods

### Data Acquisition and Processing

We collected transcriptome profiles of lung adenocarcinoma (LUAD) available in the TCGA database (https://portal.gdc.cancer.gov/) on 6 July 2021.

Preprocessed and aligned RNA-Seq samples were downloaded by selecting HTSeq-Counts as the workflow type on the portal. Clinical and pathological information related to the TCGA–LUAD cohort was retrieved from the cBioportal website (https://www.cbioportal.org) with the “cdgsr” package (Cerami et al., 2012; Jacobsen, 2015).

Meanwhile, we collected two microarray datasets and corresponding clinical information, including GSE68465 and GSE72094, from Gene Expression Omnibus using the “GEOquery” package (https://www.ncbi.nlm.nih.gov/geo/) (Davis and Meltzer, 2007). These two affymetrix microarrays were preprocessed using the RMA method (R package “affy”) (Gautier et al., 2004).

Removing samples without overall survival (OS) information or with an OS time of 0 and converting the TNM stage to AJCC staging groups, the TCGA–LUAD cohort (N = 306) was used as the training cohort, whereas GSE68465 (N = 441) and GSE72094 (N = 398) were used as the validation cohorts (Supplementary Table S1).

**FIGURE 1:**
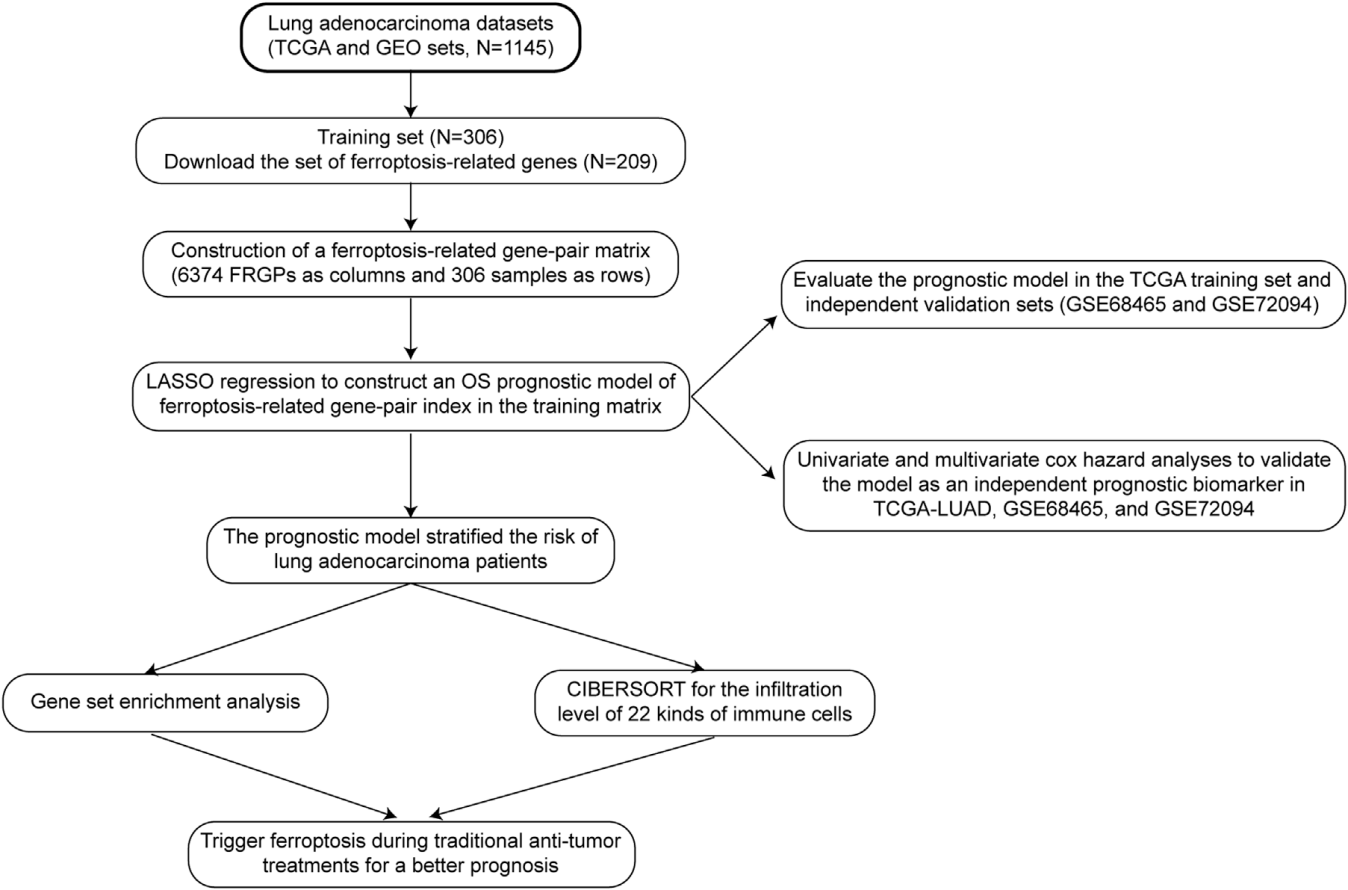
Data processing and research process.

The specific data processing and research flow are shown in Figure 1.

### Construction of the Training Matrix

One ferroptosis-related gene set was downloaded from FerrDb (http://www.zhounan.org/ferrdb/index.html), the world’s first database of ferroptosis regulators, markers, and associations (Zhou and Bao, 2020). There were 108 drivers annotated as genes that promoted ferroptosis, 69 suppressors that prevented ferroptosis, and 111 markers that indicated the occurrence of ferroptosis. Removing multi-annotated genes and selecting ferroptosis-related genes measured by using all the three cohorts, 209 genes were included in the ferroptosis-related gene set (Supplementary Figure S1).

The mean expression value of replicated genes was calculated. We defined the combination of two ferroptosis-related genes (FRG-1 and FRG-2) as a ferroptosis-related gene pair (FRGP). In every specific sample, 209 FRGs were compared by the gene expression level with each other to build 21736 FRGPs and generate a score for each FRGP. An FRGP score of 1 was assigned if FRG-1 was less than FRG-2. Otherwise, the FRGP score was 0. This gene-pair-based approach calculated the FRGP score based totally on the gene expression value of each individual sample and could be applied without normalization.

To reduce biases and be meaningful for subsequent analyses, some FRGPs counting 0s or 1s in more than 80% samples were filtered out. Finally, we got 6374 FRGPs as columns and 306 samples as rows to form the training matrix.

### Prognostic Ferroptosis-Related Gene-Pair Index Signature Construction

The least absolute shrinkage and selection operator (LASSO) is a statistical method to reduce data dimensionality. We applied the LASSO regression operation with the R package “glmnet” and “survival” in the TCGA FRGP matrix to construct a prognostic ferroptosis-related gene-pair index (FRGPI)(Simon et al., 2011). Insignificant variables whose coefficients became zero and any collinear variables were removed. 10-fold cross validation (CV) divided data into ten equal parts, nine parts as the training set and the remaining one part as the validation part. When partial likelihood deviance was the smallest, we got the minimum of lambda and nine gene pairs as our best FRGPI model. The prognostic index signature is expressed as FRGPI risk score = ∑n_i_ (FRGP_i_ *coef_i_) (*i* = 1,2,3 … … 9, *n* = 9, where *n* is the number of FRGPs, FRGPi is the score (0 or 1) of the *i*th FRGP, and coefi is the regression coefficient of the *i*th FRGP).

### Validation of FRGPI as a Prognostic Biomarker

First, we used the R package “survivalROC” to draw ROC curves and calculated the AUC values in the training and validation cohorts. AUC values greater than 0.5 and closer to 1 indicated the prognostic ability of FRGPI.

Second, we used the Kaplan–Meier method to compare survival outcomes between high- and low-FRGPI risk score groups in training and validation cohorts. The optimal cut-off value, determined based on the best balance of sensitivity and specificity to achieve the best AUC in the training cohort, was investigated using the ROC curves with the R package “survivalROC,” and “survminer” (Weiss et al., 2003).

### Validation of FRGPI as an Independent Prognostic Factor

After verification of the FRGPI significantly stratifying patients into low- and high-risk groups, we performed univariate and multivariate Cox hazard analyses to validate FRGPI as an independent prognostic factor. The hazard ratio (HR) in survival analyses less than 1 meant that the presence of the factor was protective, whereas the hazard ratio more than 1 was harmful.

### Analysis of Differentially Expressed Ferroptosis-Related Genes and *MKI67* Between High- and Low-FRGPI Groups

After constructing and validating FRGPI, we compared the expression of ferroptosis-related genes between high- and low-FRGPI groups in the TCGA cohort and GEO validation cohorts, using the Wilcoxon rank-sum test and the reshape2 package (Wickham, 2007). Meanwhile, we drew the boxplots for visualization using the ggplot2 package (Wickham, 2009).In addition, we compared *MKI67* expression between high- and low-FRGPI groups in the three cohorts. *MKI67* encodes a nuclear protein Ki-67, which is a commonly used marker for cell proliferation.

### Gene Set Enrichment Analysis for Kyoto Encyclopedia of Genes and Genomes

Gene set enrichment analysis (GSEA) determines whether the gene sets, not single genes, present differences between different biological status groups and verifies that the gene sets are enriched in one specific clinical group (Subramanian et al., 2005). The gene sets are predefined by previous experiments and function annotations.

We chose gene sets from the Kyoto Encyclopedia of Genes and Genomes (KEGG) pathway which is a collection of pathway maps representing molecular interactions, reactions, and relation networks (Kanehisa et al., 2016).

We performed GSEA–KEGG analyses and drew enrichment plots in the TCGA cohort and GEO validation cohorts, using “c2.cp.kegg.v7.4.entrez.gmt” and the R package “clusterProfiler,” and “ggplot2,” defining p value < 0.05 and q value < 0.05 as the filtering criteria (Yu et al., 2012).

### Calculation of the Infiltration Level of 22 Kinds of Immune Cells

Cell-type Identification By Estimating Relative Subsets Of RNA Transcripts (CIBERSORT) is a deconvolution method for characterizing the cell composition of complex tissues from gene expression profiles (Newman et al., 2015). We made 22 kinds of immune cells as the target characterizing composition. We operated the “CIBERSORT” algorithm with the “Leukocyte signature matrix” (Newman et al., 2015). Based on the composition of 22 kinds of immune cells, we screened out what kind of immune cells differently infiltrated between high- and low-FRGPI groups.

### Statistical Analysis

All statistical analyses were based on R Programming Language software (Rx64 3.3.3). The online website “www.genome.jp/kegg/” was used for GSEA–KEGG gene sets. The website “cibersortx” offered thoughts for CIBERSORT analyses in R software.

## Results

### Prognostic Ferroptosis-Related Gene-Pair Index Construction

First, we downloaded one ferroptosis-related gene set including 209 genes. Two genes as one couple, any couple of the 209 genes could be created. So, 209 genes formed 21736 FRGPs in every individual sample. To calculate each FRGP in every sample, if the FRG-1 value was less than the FRG-2 value, the FRGP score was 1. Otherwise, the FRGP score was 0. Then, we filtered out FRGPs with constant values (0 or 1) in TCGA or GEO datasets. Finally, we got 6374 FRGPs in every individual sample. In TCGA training cohort, we used 306 samples as rows and 6374 FRGP scores as columns to make up the training FRGP matrix.

**FIGURE 2:**
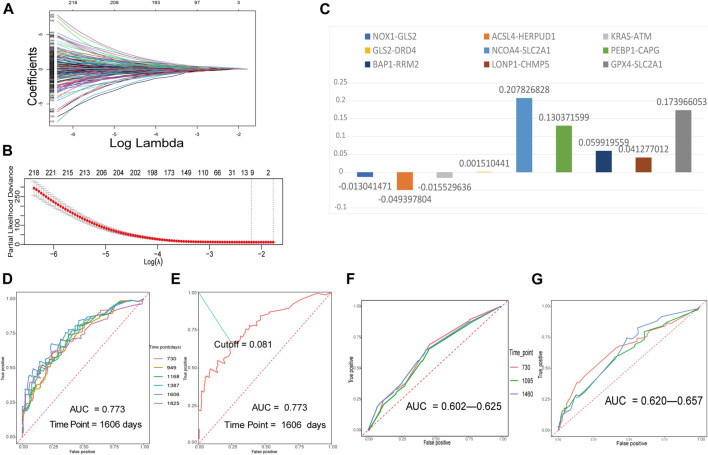
Building a ferroptosis-related gene-pair index (FRGPI) in the training set and verifying FRGPI in the ROC curves. **(A)** The diagram of lambda and coef. **(B)** Performing 10-fold CV to calculate the partial likelihood deviance corresponding to different models. The deviance was the smallest when nine gene pairs were included. The minimum of lambda was 0.1115. **(C)** Our prognostic FRGPI was made up of nine FRGPs and corresponding coef values. **(D)** The time-dependent ROC curves of the training set. **(E)** We defined the optimal cut-off 0.081 in the training set curve with the best AUC and “1606” as the time point. **(F,G)** Time-dependent ROC curves of validation cohorts.

The training FRGP matrix was used for evaluating the relationship between FRGPs and overall survival rates applied using the LASSO regression operation. The lambda and coef diagrams of FRGPs (Figure 2A) were plotted using the LASSO algorithm. With the increase in the lambda value, the coefficients of some FRGPs decreased to zero, which meant that the scores of these FRGPs did not affect the model. We then used 10-fold CV to calculate the partial likelihood deviance of the model (Figure 2B). The minimum deviance exported the best model. The best model included nine gene pairs. Finally, we constructed a prognostic FRGPI signature with nine FRGPs and the corresponding coef values (Figure 2C).

### Verification of FRGPI as a Prognostic Biomarker

The time-dependent ROC curves with AUC values of the training and validation cohorts are all presented in Figures 2D,F,G. All the AUC values were more than 0.5 and even greater than 0.7 in training and validation cohorts, indicating that FRGPI had a favorable prognostic ability.

In the training cohort, the AUC value reached 0.773 for 1606 days. We defined the optimal cut-off 0.081 in the curve with the best AUC and “1606” as the time point (Figure 2E). 0.081 was used as a cut-off for FRGPI to stratify patients into the low- or high-FRGPI risk score group.

**FIGURE 3:**
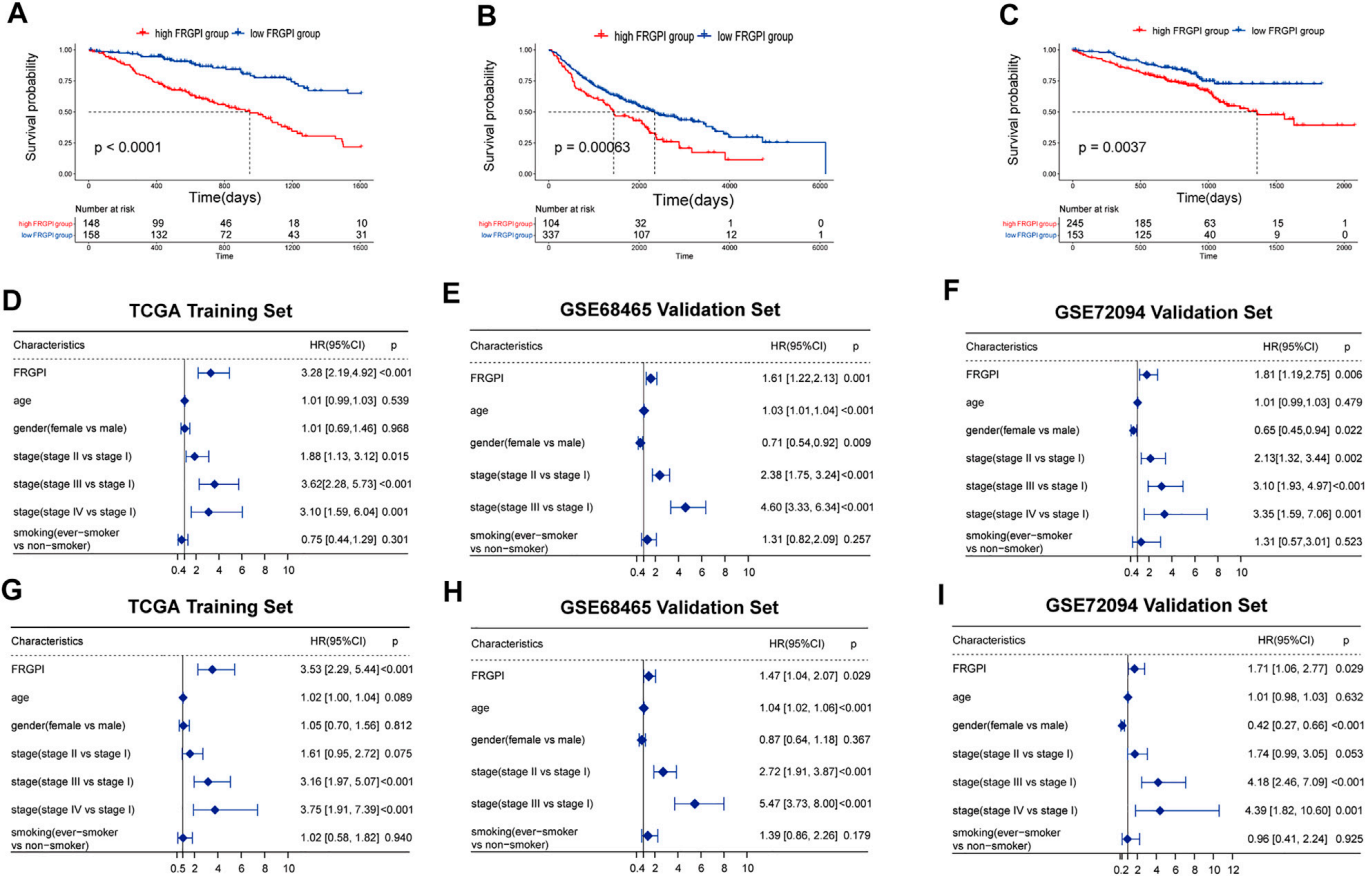
Verification of FRGPI as an independent prognostic biomarker. **(A–C)** Kaplan–Meier curves between high- and low-risk FRGPI groups in training and validation sets. **(D–F)** Univariate Cox analyses of the three cohorts. **(G–I)** Multivariate Cox analyses of the three cohorts.

We then performed Kaplan–Meier curves between high- and low-FRGPI groups. All three curves showed that the low-FRGPI group had a significantly better prognosis than the high-FRGPI group (*p < 0.01*, Figures 3A–C).

To further validate FRGPI as a prognostic biomarker, the low-FRGPI group also had significantly better prognoses than the high-FRGPI group for 2 and 5 years (*p < 0.05*, Supplementary Figures S2A–F). In the early-stage LUAD, all the Kaplan–Meier curves showed that the low-FRGPI group had a significantly better prognosis (*p < 0.01*, Supplementary Figures S2G–I). Additionally, ever-smokers with low FRGPI scores owned a better prognosis (*p < 0.05*, Supplementary Figure S3). Overall, FRGPI successfully stratified the risk of LUAD patients in all the training and validation sets.

### Validation of FRGPI as an Independent Prognostic Factor

Univariate regression results showed that FRGPI was statistically significant in the training and validation cohorts (*p < 0.01*, Figures 3D–F). The multivariate regression results showed that FRGPI was an independent prognostic factor in all three cohorts (*p < 0.05*, Figures 3G–I). All univariate and multivariate results revealed that the high-FRGPI group matched with a worse prognosis, with the HR and 95% confidence interval HR of FRGPI more than 1.

In the multivariate regression, it was shown that our FRGPI could be as good as stage III vs. stage I in stratifying patients. Patients whose cancer has progressed to stage III, especially stage IIIB, could hardly get radical surgical therapy. The median PFS of these patients is about 10 months (Ryan et al., 2019).

### Analysis of Differentially Expressed Ferroptosis-Related Genes and *MKI67* Between High- and Low-FRGPI Groups

**FIGURE 4:**
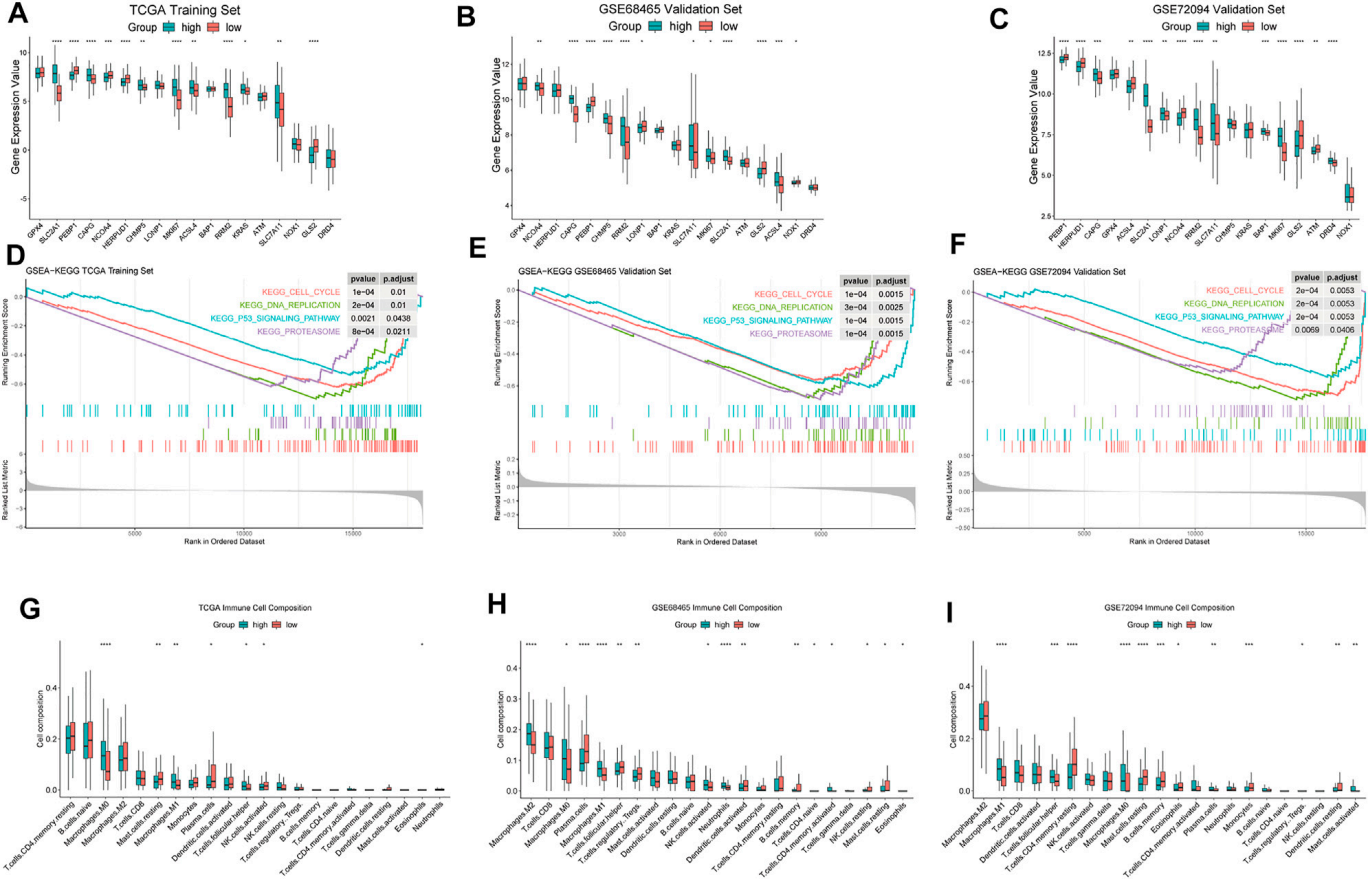
Differences of biological characteristics between high- and low-FRGPI groups. We used the following convention for symbols indicating statistical significance: *: *p < 0.05*, **: *p < 0.01*, ***: *p < 0.001*, and ****: *p < 0.0001*. **(A–C)** ferroptosis-related genes and *MKI67* for the expression level comparisons in the three cohorts. **(D–F)** Enriched GSEA–KEGG pathways in the three cohorts. **(G–I)** Infiltration levels of 22 kinds of immune cells in the three cohorts.

Our FRGPI included 16 ferroptosis-related genes. In addition, SLC7A11 as the main target of FINs was added. We compared the expression of 17 ferroptosis-related genes and *MKI67* between high- and low-FRGPI groups (*p < 0.05*, Figures 4A–C). In training and validation cohorts at the same time, solute carrier family 2 member 1 (*SLC2A1*), gelsolin-like actin-capping protein (*CAPG*), ribonucleotide reductase regulatory subunit M2 (*RRM2*), *SLC7A11*, and *MKI67* were significantly up-regulated in the high-FRGPI group. On the contrary, we found *GLS2* and phosphatidylethanolamine-binding protein 1 (*PEBP1*) were down-regulated in the high-FRGPI group in all three cohorts. The high-FRGPI group was marked with significantly higher *MKI67* expression and exhibited higher cancer proliferation potential.

### GSEA Based on High- and Low-FRGPI Groups

FRGPI separated patients into high and low groups. The differences of enriched GSEA–KEGG pathways between the two groups are shown in Figures 4D–F. There was no enriched KEGG pathway in the low-FRGPI group in the training and validation cohorts at the same time. Conversely, we found that “KEGG CELL CYCLE,” “KEGG DNA REPLICATION,” “KEGG PROTEASOME,” and “KEGG P53 SIGNALING PATHWAY” were significantly enriched in the high-FRGPI group in all three cohorts (*p < 0.05*, Figures 4D–F). We inferred that the consistently enriched pathways in the high-FRGPI group in part played important roles in the worse prognosis.

### High-FRGPI Group Presented Higher M1 Macrophage Infiltration

The differences of infiltration levels of 22 kinds of immune cells are shown in Figures 4G–I. We found that M1 macrophages were significantly up-regulated in the high-FRGPI group in all three cohorts; however, activated CD4^+^ cells, CD8^+^ cells, dendritic cells, plasma cells, and natural killer cells did not present different infiltration levels. There were few differences in adaptive immune responses between high- and low-FRGPI groups.

## Discussion

We built one prognostic model in lung adenocarcinoma consisting of nine ferroptosis-related gene pairs. The 9 gene pairs include 16 individual genes which participate in multiple crucial molecular mechanisms of ferroptosis and tumorigenesis. Then, we found that *SLC2A1*, *PEBP1*, *CAPG*, *RRM2*, *SLC7A11*, and *GLS2* differentially expressed between high- and low-FRGPI groups in all three datasets. Down-regulation of *SLC2A1* can suppress the progression of lung adenocarcinoma (Wang et al., 2017). PEBP1 binds to ALOX15, which is essential for ferroptosis, to promote lipid peroxidation and induce ferroptosis (Wenzel et al., 2017). CAPG and RRM2, could inhibit ferroptosis after stimulation of erastin (Zhang et al., 2019).

Both glutaminase 1 (GLS1, kidney type) and glutaminase 2 (GLS2, liver type) catalyze the conversion of glutamine into glutamate. Nevertheless, only GLS2 is involved in the up-regulation of ferroptosis by inhibiting the production of GPX4 and promoting downstream lipid ROS manufacture (Lukey et al., 2019; Tang et al., 2021). Meanwhile, increased nuclear translocation of GLS2 has been reported to stop the cell cycle at the G2/M stage to prevent proliferation (El-Deiry, 2016). Overexpression of *GLS2* in human lung, liver, and colon cancer cells has been proved to induce significant inhibitions in tumor growth and proliferation (Suzuki et al., 2010). Therefore, as shown in our KEGG results, “KEGG CELL CYCLE” was not enriched in the group in which *GLS2* was up-regulated. Overall, up-regulation of *GLS2* in the low-FRGPI group might be associated with promoting cancer ferroptosis and preventing tumor proliferation.

The GSEA–KEGG results revealed that the four pathways, “KEGG CELL CYCLE,” “KEGG DNA REPLICATION,” “KEGG PROTEASOME,” and “KEGG P53 SIGNALING PATHWAY,” were enriched in the high-FRGPI group. “Cell cycle” and “DNA replication” gene sets were associated with cell proliferation and cancer aggressiveness. At the same time, the high-FRGPI group was also marked with higher *MKI67* expression. The high-FRGPI group suffered more risk of tumor progression and might have a worse prognosis.

As previously observed, a high infiltration level of M1 macrophages might be associated with a better survival outcome in NSCLC patients (Ma et al., 2010). Contradictorily, in our study, the high-M1/FRGPI group demonstrated a worse outcome in all three datasets. This, at least, was not a coincidence. Recent reports in breast cancer showed that “M1” high tumors were definitely associated with more aggressive clinical features (Lu and Ma, 2020; Oshi et al., 2020). In our research, we also detected that the high-M1/FRGPI group presented a higher *MKI67* expression and enriched “cell cycle” and “DNA replication” gene sets, which meant that the high-M1/FRGPI group might have more aggressive cancer cells and advanced cell proliferation. Meanwhile, our CIBERSORT results revealed that the high-M1/FRGPI group did not present favorable immune activities to fight with aggressive cancer cells. Overall, the anti-cancer tumor immune microenvironment could not counterbalance the biologically aggressive features of the high-M1/FRGPI group, possibly leading to the worse survival outcome of the high-M1/FRGPI group.

We detected that *SLC7A11* was up-regulated in the high-FRGPI group. Cystine transporter xCT encoded by *SLC7A11* exports intracellular glutamate and imports extracellular cystine for glutathione biosynthesis and downstream GPX4 to reduce lipid peroxidation. Class I FINs aim at inhibiting SLC7A11 activity to trigger ferroptosis in cancer cells. In addition, class I FINs, such as erastin, synergistically enhance the anti-tumor effect of classical cisplatin chemotherapy in NSCLC (Guo et al., 2018). Superabundant antioxidants in cancer build a huge obstacle to radiotherapy. Class I FINs deplete GPX4 to promote lipid peroxidation and enhance radio sensitivity. In treating lung adenocarcinoma, erastin and x-ray irradiation reinforce each other (Shibata et al., 2019). In brief, the up-regulation of *SLC7A11* in the high-FRGPI group with a worse prognosis indicated one treatment strategy to particularly inhibit SLC7A11 and activate ferroptosis. The strategy focused on using class I FINs to inhibit SLC7A11, induce ferroptosis, and synergistically work together with traditional chemotherapy and radiotherapy for the high-FRGPI group to gain a better prognosis.

In conclusion, we built a robust gene-pair prognostic model of lung adenocarcinoma on the basis of ferroptosis mechanisms. We applied this model to stratify patients into low- and high-FRGPI groups. Moreover, we explored the differences of the biological pathways and tumor immune microenvironment between high- and low-FRGPI groups. Previously, based on candidate prognostic genes, Li et al. (2019), Al-Dherasi et al. (2021), and Liu et al. (2021) identified prognostic signatures in lung adenocarcinoma. These models included 16 genes, 7 genes, and 4 genes, respectively. Liu et al. (2020) identified a 14-gene signature in lung adenocarcinoma based on differentially expressed genes between tumor and normal tissues. However, this signature was short of validations in independent datasets. Liang et al. (2021) also established one 7-gene prognostic model based on ferroptosis-related genes in lung adenocarcinoma. The AUC of 5-year survival was 0.709 in Liang et al.’s training cohort. Our signature utilized gene pairs to overcome technical problems regarding the comparison between different datasets. The AUC of our signature was 0.773 in the training cohort. In addition to the KEGG pathway enrichment analysis in Liang et al.’s model, we focused on the differentially expressed genes and tumor immune microenvironment. We supposed SLC7A11 as the target of FINs might have something to do with outcomes and guide us to trigger ferroptosis during traditional treatments for better prognoses.

Although we verified FRGPI as an independent prognostic biomarker, our study exposed limitations. Our FRGPI was based on large-scale network datasets and lacked additional local patient data. We used ferroptosis-related gene pairs as FRGPI’s components to avoid normalization, but still faced complex intrinsic and extrinsic interference factors which might affect FRGPI accuracy. Meanwhile, the specific functions and biological pathways of genes and gene pairs in FRGPI need further investigation. Moreover, prospective studies and experiments are required for further validations of FRGPI and careful considerations of FRGPI for individual therapies.

## Data Availability

The original contributions presented in the study are included in the article/Supplementary Material, further inquiries can be directed to the corresponding author.
