# Supplementary material for: One Ferroptosis-Related Gene-Pair Signature Serves as an Original Prognostic Biomarker in Lung Adenocarcinoma
Source: Front Genet. 2022 Mar 16;13:841712. doi: 10.3389/fgene.2022.841712 (PMC8965883; doi:10.3389/fgene.2022.841712)
Supplement: Supplementary file 1 [file DataSheet1.pdf]

Table S1 | Detailed patient clinical characteristics

Characteristics	Training set	Validation set 1	Validation set 2
Source	TCGA-LUAD	GSE68465	GSE72094
Sample size	306	441	398
Platform	RNA-seq	Affymetrix Human Genome U133A Array	Rosetta/Merck Human RSTA Custom Affymetrix 2.0 microarray
**AJCC stage**			
Stage I	160	275	254
Stage II	67	95	67
Stage III	56	68	57
Stage IV	18	-	15
Unknow	5	3	5
**Age group**			
⩽65	151	230	118
> 65	148	211	280
Unknow	7	0	0
**Smoking history**			
Non-smoker	37	48	30
Ever-smoker	250	297	298
Unknown	19	96	70
**Gender**			
Male	155	222	176
Female	151	219	222
**Survival status**			
Alive	187	206	285
Dead	119	235	113

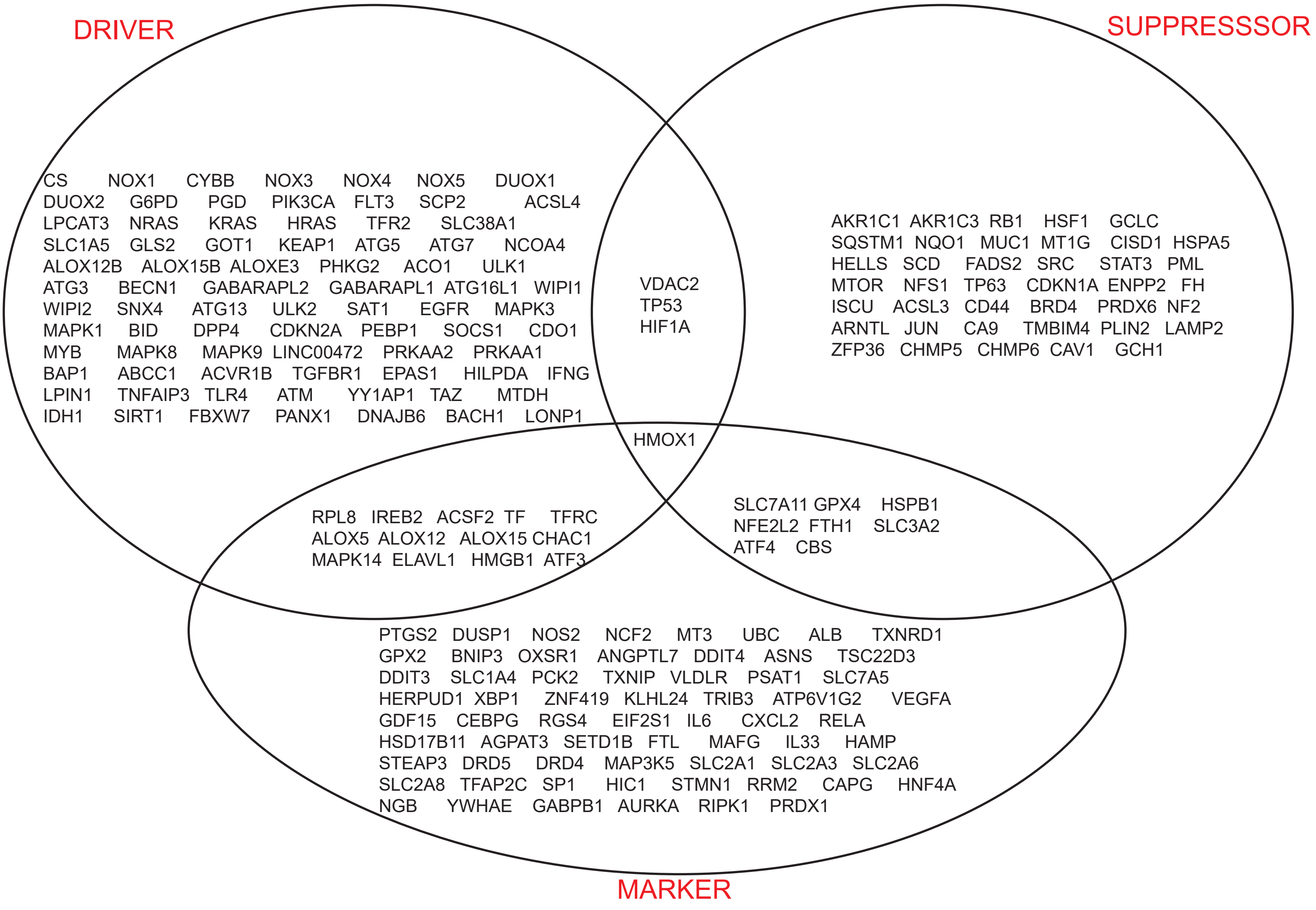

Figure S1 | 209 genes were included in the ferroptosis-related gene set.

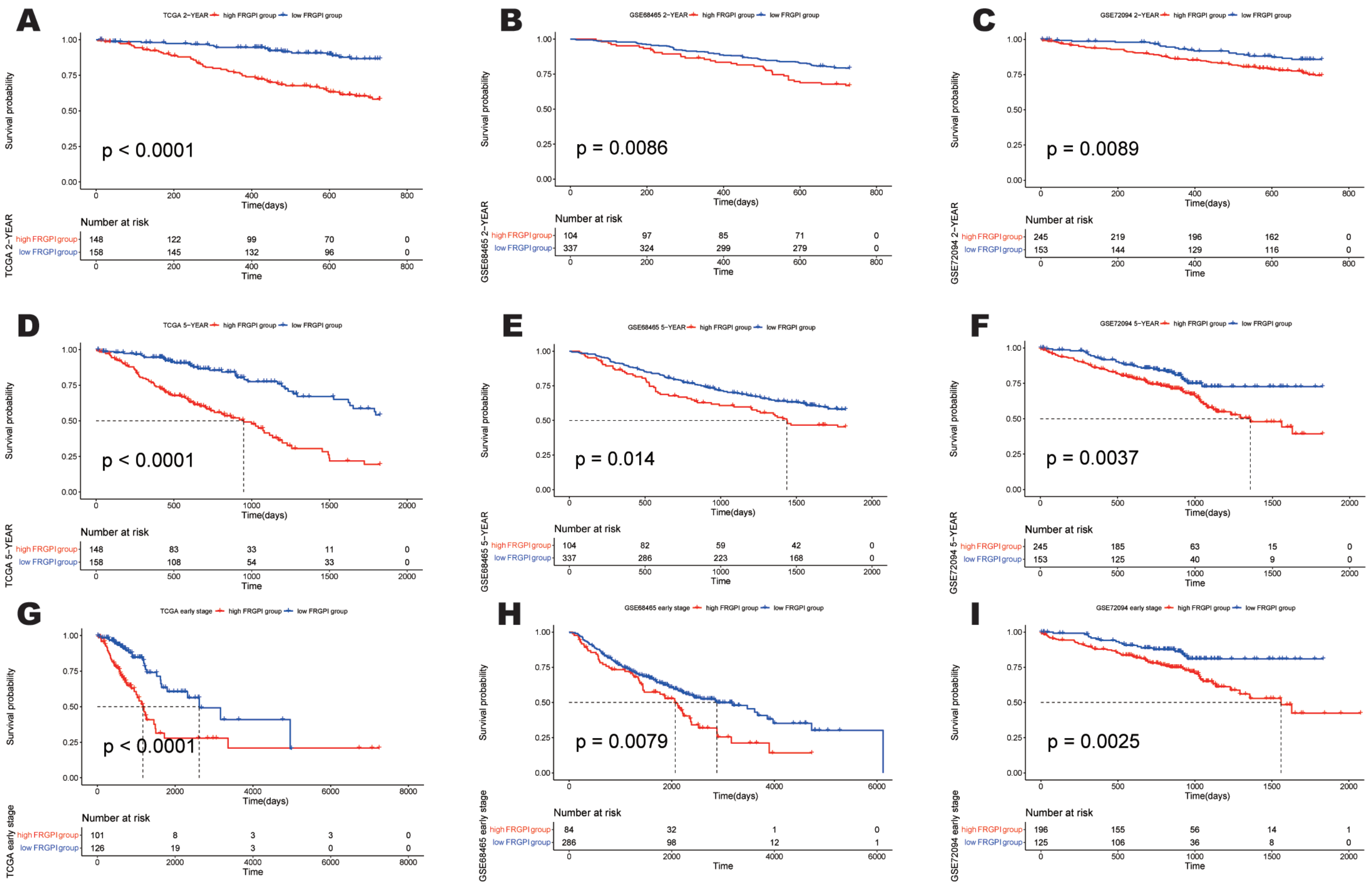

Figure S2 | The low FRGPI group had a significantly better prognosis than the high FRGPI group. (A-C) For 2 years (D-F) For 5 years (G-I) In the early stage lung adenocarcinoma

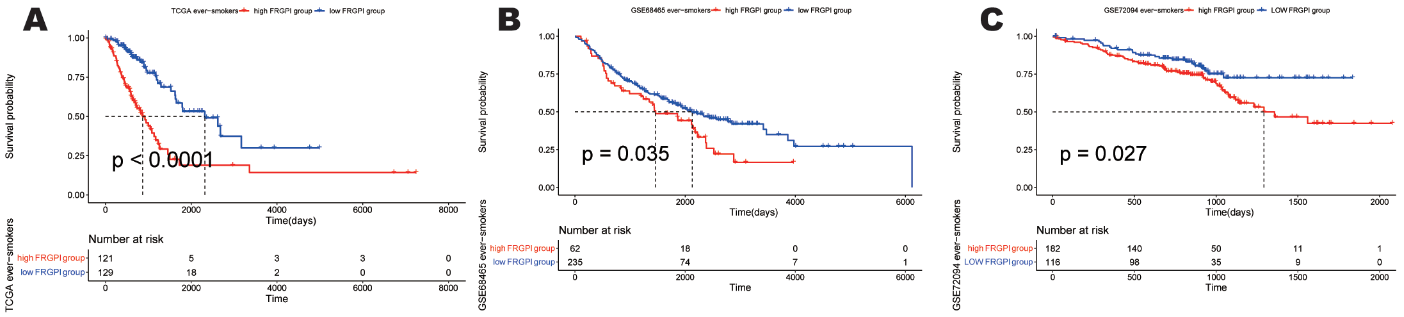

Figure S3 | Ever-smokers with low FRGPI owned a better prognosis.
